# Knowledge, attitudes, and practices of radiology practitioners in Saudi Arabia toward the use of gonad shields during fluoroscopy-guided interventional radiography

**DOI:** 10.3389/fmed.2025.1655457

**Published:** 2025-09-25

**Authors:** Mamdouh S. Alenezi, Abdulaziz M. Alshammari, Mazin B. Hassib

**Affiliations:** Department of Diagnostic Radiology, College of Applied Medical Sciences, University of Hail, Hail, Saudi Arabia

**Keywords:** fluoroscopy, interventional radiology, gonad, occupational hazards, radiation exposure, healthcare attitudes

## Abstract

**Background:**

The utilization of gonad shields in interventional radiology (IR) settings plays a pivotal role in minimizing radiation risk for practitioners.

**Purpose:**

This study aims to assess the impact of experience, practitioners’ opinion, education in radiation protection, practitioners’ gender, workload, and educational level on the use of gonad shields.

**Methods:**

This cross-sectional questionnaire study comprised six hypotheses that were designed to fulfill the study’s aim. Normality was assessed using skewness and kurtosis values within a specified threshold. Categorical variables were assessed using cross-tabulation and the chi-square (χ^2^) test of independence. A *p*-value of <0.05 was considered statistically significant.

**Results:**

The study included 527 participants (307 female and 220 male). There was a significant inverse association between years of experience and the frequency of gonad shield use (*p* < 0.05). A direct relationship was observed between practitioners’ opinions on the importance of gonad shields and shield usage frequency (*p* < 0.05). It was clear that attending radiation protection training was more likely to encourage practitioners to follow gonad shielding protocols (*p* < 0.05). No significant effect of gender and gonad shield usage was observed (*p* = 0.086). Practitioners with higher annual caseloads (>200 cases/year) reported more consistent use of gonad shields (*p* < 0.05).

**Conclusion:**

Practice length, but not workload, is inversely related to IR staff’s attitude toward gonad shield use. Continuous radiation protection training is crucial for improving IR staff commitment to safety standards. No impact of gender on gonad shield usage was observed. Targeted refresher training, reinforcement of local guidance, and ensuring shield availability/workflow integration may strengthen occupational radiation-safety adherence in IR units.

## 1 Introduction

Charles Dotter first conceptualized interventional radiology (IR) in 1963 (1), with the first arterial angioplasty performed in 1964 on an 82-year-old woman using a guide wire and coaxial Teflon catheters (2). This marked the successful introduction of a new minimally invasive medical imaging and therapy technology that uses ionizing radiation for diagnosis and treatment (3).

Fluoroscopy-guided interventions necessitate the practitioner’s proximity to the patient for real-time visualization and guidance; therefore, maintaining distance, a primary radiation safety measure, is not always feasible. Although radiation offers significant benefits in diagnosing and treating patients, it also carries inherent risks for healthcare practitioners, including cancer and reproductive hazards (4–7). Consequently, IR practitioners should carefully consider the balance between the merits of high-fidelity imaging and the risks inherent to radiation exposure to maintain radiation doses as low as reasonably achievable (ALARA) (8). Contemporary studies report disparities in the application of radiation-protection behaviors, thus reinforcing the need for operator-focused shielding within ALARA-based programs and local protocols (9–16).

For IR practitioners, protective shields help reduce occupational radiation exposure during fluoroscopy-guided IR. Gonad shields are designed to minimize radiation exposure to sensitive reproductive organs, lowering the risk of genetic damage and reproductive problems; this is crucial in interventional radiology, especially for men, because of the vulnerability of male reproductive tissue to radiation (17, 18). However, evidence on operators’ actual use of gonad shields remains limited, particularly in Saudi Arabia. Prior surveys have focused on general radiation safety knowledge or patient shielding rather than operator gonad shielding. Few studies measure how other factors, such as training, years of IR experience, gender, and annual workload, relate to practice in real clinical environments (9, 10, 12–14).

To address this gap, we conducted a multi-institutional survey of IR practitioners in Saudi Arabia focusing on knowledge, attitudes, and practices (KAP) regarding operator gonad shields and potential determinants, such as training, experience, workload, education, availability, local guidance, and gender. Unlike previous studies that concentrated on broad safety education or patient safeguards, our research quantifies the use of operator shields by the interventional radiology workforce in Saudi Arabia.

## 2 Materials and methods

### 2.1 Study design and setting

This analytical cross-sectional study was conducted across multiple healthcare institutions in Saudi Arabia. The study aims to identify the factors influencing gonad shield utilization during fluoroscopy-guided IR procedures. A structured, self-administered electronic questionnaire was distributed to 527 IR practitioners. Data were collected between September 2024 and February 2025.

### 2.2 Participations

Eligible participants were practicing radiology professionals involved in fluoroscopy-guided IR, including radiologists, IR fellows/registrars, and technologists/radiographers, who were working in Saudi Arabia during the study period. Individuals outside IR, those not engaged in fluoroscopy-guided procedures, and incomplete submissions were excluded. A total of 527 practitioners completed the questionnaire.

Using a conservative single-proportion approach (*p* = 0.50, margin of error d = 0.05, Z = 1.96), the minimum required sample size (N) was 384, based on the Charan and Biswas formula. The actual sample size (*N* = 527) exceeds this requirement, resulting in narrower confidence intervals for prevalence/awareness estimates and supporting the planned subgroup and multivariable analyses. The formula and derivation are shown in Supplementary file.

### 2.3 Data collection

Using a structured self-report questionnaire, data were collected through institutional contacts and professional networks. Participation was voluntary and anonymous, and questionnaires were specifically designed to capture demographics, professional profile, experience, knowledge, attitudes, departmental guidance, and practices related to gonad shield use in IR.

### 2.4 Research instrument

The questionnaire was developed through a targeted literature review and researcher-created items tailored to fluoroscopy-guided IR. It comprises three sections: informed consent, participant demographics, and IR practices with gonad shield use (Supplementary Table S1). We hypothesized that viewing shielding as important, alongside higher caseloads, would be linked to increased use, and greater experience would correlate with lower use. Training and local guidance would encourage greater protocol adherence, in addition to the influences of educational level and gender (Supplementary Table S2).

### 2.5 Statistical analysis

Data analysis was performed using IBM SPSS Statistics Version 20 and Microsoft Excel. The formula developed by Charan and Biswas was used to determine the sample size (Supplementary file) (19). The reliability was assessed using the Cronbach’s Alpha Reliability Test. Skewness and kurtosis values were assessed to confirm the normality of the data. Normality was assumed when the skewness and kurtosis values were within the ranges of ±2 and ±4, respectively (20). Cross-tabulation and the chi-square test of independence (χ^2^) were used to analyze categorical data and evaluate the relationships between practitioner characteristics and shielding-related outcomes (21), in addition to proportional-odds ordinal logistic regression (OLR) with a 95% confidence interval. Gender was analyzed using χ^2^ only, because the corresponding outcome was nominal and lacked an inherent order, making OLR inappropriate. Cramér’s V was used to analyze the effect size. Responses were summarized as n (%) using available-case denominators. A *p*-value of < 0.05 was considered statistically significant.

### 2.6 Ethical consideration

The Research Ethics Committee (REC) reviewed and approved this research (H-2024-407, 02/09/2024 G). Informed consent was obtained from every participant before their involvement. Participant anonymity was preserved; no names or identifying data were included.

## 3 Results

### 3.1 Participant characteristics and key responses

The study involved 527 interventional radiology practitioners (Supplementary file), of which there were 307 (58.3%) women and 220 (41.7%) men. Key shield-related variables are summarized in Table 1 (availability, use frequency, training, and local guidance). Additional demographics, including marital status, educational level, years of experience, and annual workload, are reported in the Supplementary file and Supplementary Table S1.

**Table 1:** Questionnaire responses to structured items for measuring practitioner behaviors and perspectives in gonad shield usage (*N* = 527).

No.	Survey question	Answer option	n	%
1	Are gonad shields available in your workplace?	Yes	390	74
		No	55	10.4
		I do not know	82	15.6
		Total	527	100
2	How often do you use gonad shields in interventional radiography procedures?	Always (in all cases)	231	43.8
		Often (in most cases)	96	18.2
		Sometimes (occasionally)	109	20.7
		Rarely (in few cases)	23	4.4
		Never	91	17.3
		Total	527	100
3	What is your most probable reason for not using gonad shields?	Not available	55	10.4
		Too busy to do so	420	79.7
		Not important	52	9.9
		Total	527	100
4	Which reason increases the likelihood of you using gonad shields?	Departmental protocol and/or guideline	145	27.5
		Fear and anxiety about radiation exposure	273	51.8
		Education and training	109	20.7
		Total	527	100
5	In your opinion, regardless of your practice, is it important to protect the gonads?	Yes	435	82.5
		No	28	5.3
		I am not sure	64	12.1
		Total	527	100
6	What is your opinion regarding the importance of gonad shields?	Not important	51	9.7
		Slightly important	124	23.5
		Important	352	66.8
		Total	527	100
7	Have you ever attended lecture(s) and/or training sessions on radiation protection?	Yes	374	71.0
		No	99	18.8
		I do not remember	54	10.2
		Total	527	100
8	What would be your attitude if your department discontinued gonad shield use?	I would continue using the gonad contact shield	272	51.6
		I would use a gonad shield in selective procedures	175	33.2
		I would stop using the gonad shield	80	15.2
		Total	527	100
9	Gonad shields for practitioners:	Should be used for females only	70	13.3
		Should be used for all genders	387	73.4
		Should be used for married persons only	30	5.7
		Should not be used at all	40	7.6
		Total	527	100
10	Do you follow any particular guidance on the use/non-use of gonad shields?	Yes, guided by local protocols	255	48.4
		No guided rules	108	20.5
		I do not know	164	31.1
		Total	527	100

### 3.2 Data analysis

Table 1 summarizes participants’ responses to 10 questionnaire items that cover the availability, frequency, attitudes, awareness, and institutional guidance related to gonad shield use in IR. The consistency was assessed using Cronbach’s alpha, and the Cronbach’s alpha value was 0.874 [>0.7 (22)]. Therefore, it is reasonable to state that the study questionnaire demonstrates high reliability. The normality test was performed on the items listed in Table 1, using the skewness and kurtosis thresholds (±2 and ±4, respectively). Most of the items exhibited skewness and kurtosis within the expected range, which implies a normal distribution.

**Figure 1:**
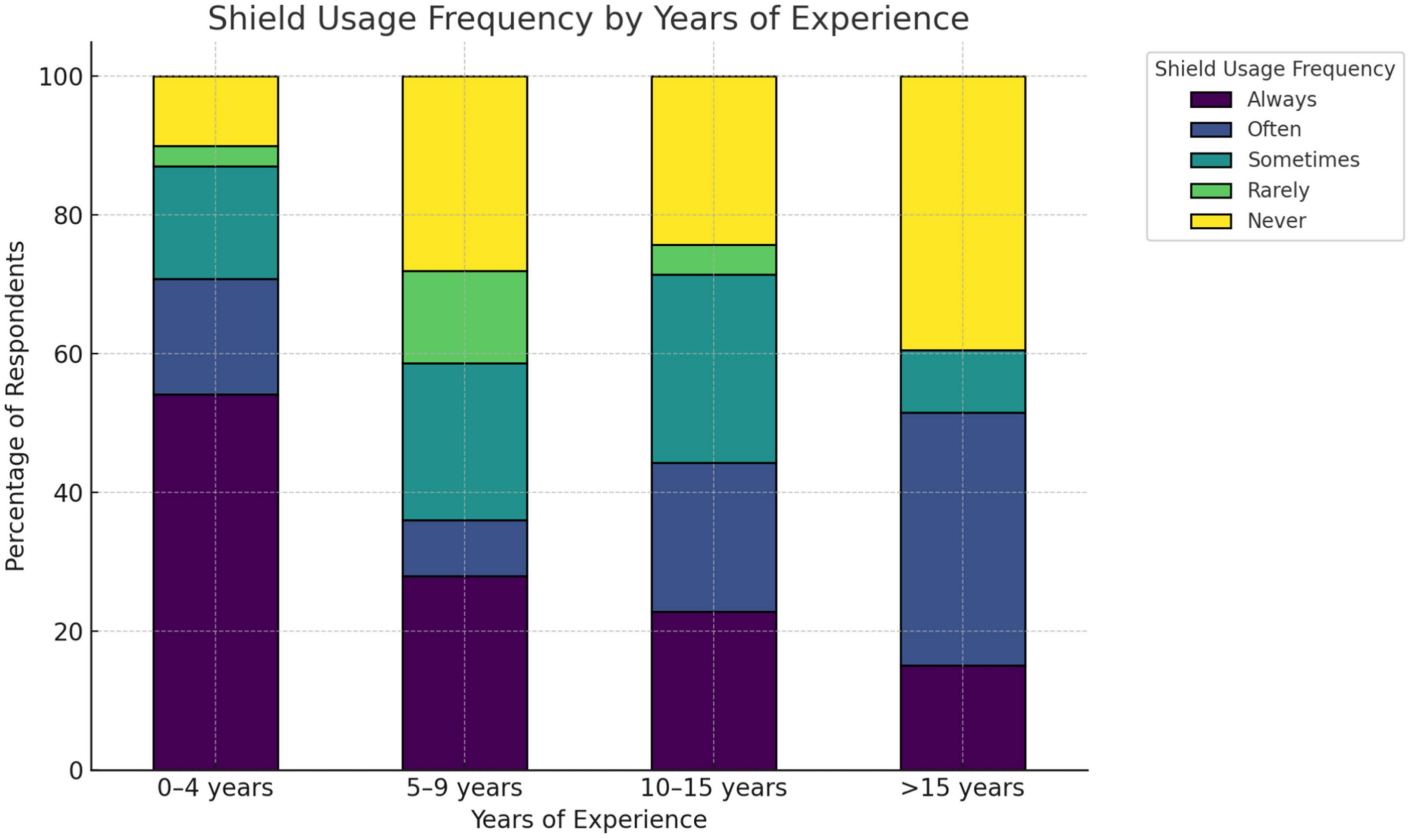
Years of experience vs. frequency of gonad-shield use. Stacked columns show column-wise percentages of usage within each experience group.

In the analysis of the effect of experience on gonad shield usage, cross-tabulation showed an apparent decline in gonad shield usage as experience increased (Figure 1). A chi-square test confirmed a statistically significant association between experience and usage frequency, χ^2^ = 88.83, *p* = 8.3 × 10^−14^ (*p* < 0.05), Cramér’s V = 0.24. Years of experience were inversely related to gonad shield usage frequencies. Ordinal logistic regression further demonstrated that all experience groups of more than 0–4 years were significantly less likely to report higher usage frequencies (*p* < 0.05). The regression coefficients and confidence intervals demonstrate the decreasing likelihood of consistent shield use with increasing years of experience (Supplementary Figure S1).

**Figure 2:**
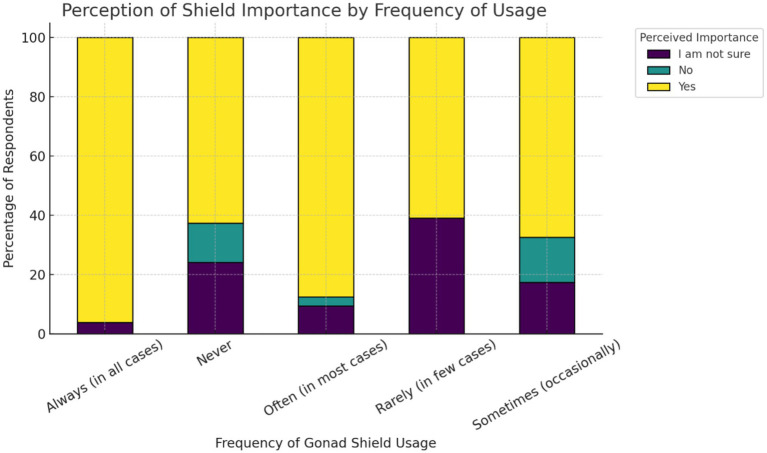
Stacked percentage bar chart illustrates the perceived importance of shielding by frequency of usage.

In terms of attitudinal salience, recognizing the need to protect the gonads strongly correlates with more frequent use (Figure 2). Respondents who were unsure (and, to a lesser extent, those not in favor) clustered in lower-use categories: χ^2^ = 94.19, *p* = 6.53 × 10^−17^ (<0.05), Cramér’s V = 0.30. The relationship is moderate in strength and is supported in direction by OLR (Supplementary Figure S2).

**Figure 3:**
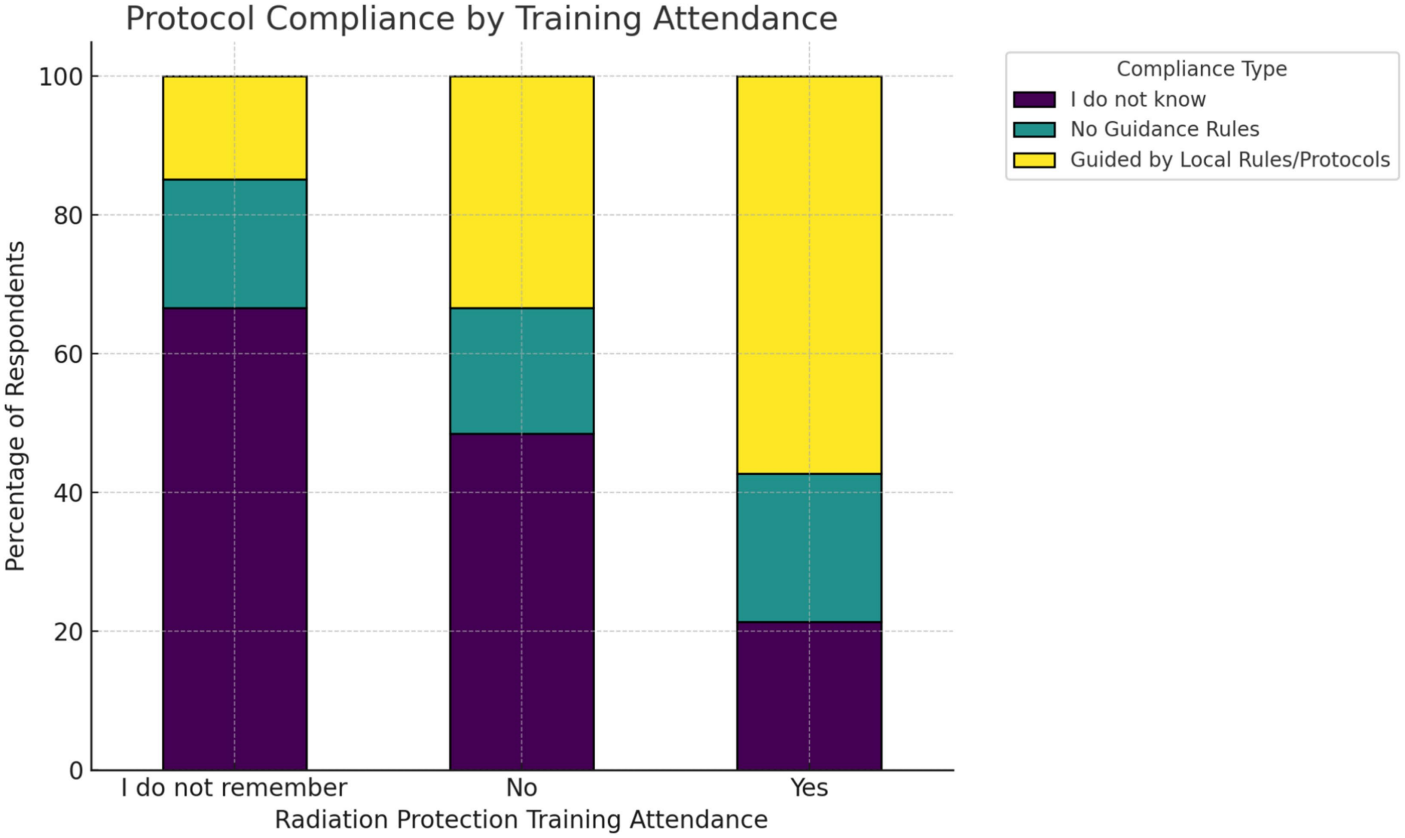
The stacked percentage bar chart displays the distribution of protocol compliance as a function of radiation protection training attendance.

Regarding radiation protection awareness and gonad shield use, cross-tabulation revealed that practitioners who reported attending radiation protection training were significantly more likely to follow local gonad shielding protocols (Figure 3).

Furthermore, among those trained, more than half indicated adherence to official guidance, whereas those who did not attend or could not recall attending training showed greater uncertainty or lack of awareness of protocols. The chi-square test confirmed a strong association between training attendance and compliance behavior (χ^2^ = 66.65, df = 4, *p* = 1.16 × 10^−13^ (<0.0001), Cramér’s V = 0.25). This small to moderate correlation corresponds with higher adjusted odds of compliance among trained practitioners (Supplementary Figure S3).

**Figure 4:**
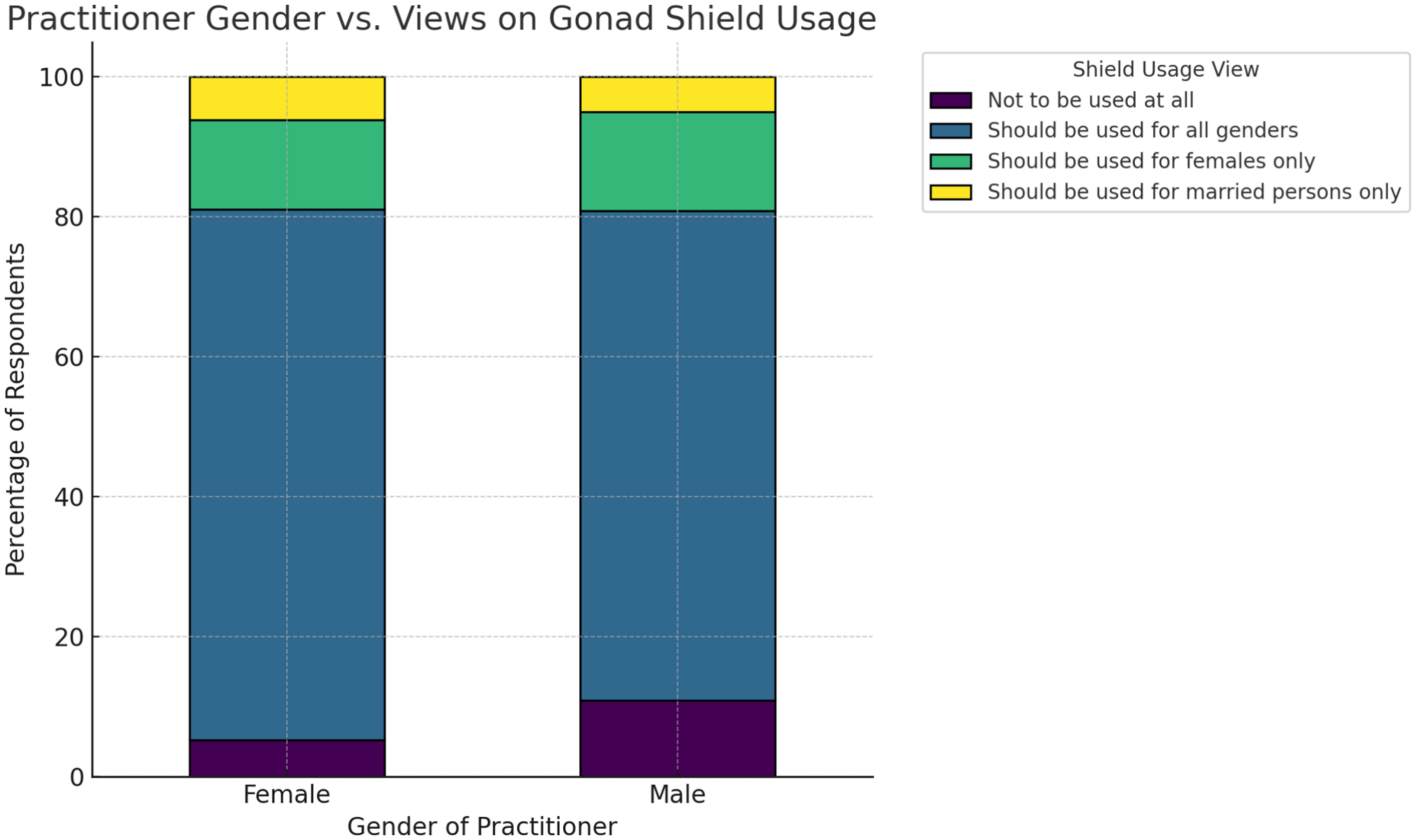
The stacked percentage bar chart of gonad shielding views by practitioners’ gender.

The analysis of gender type and adherence to gonad shield usage through cross-tabulation showed that both male and female practitioners primarily favor the use of gonad shields (Figure 4). The chi-square test indicated no statistically significant association between practitioner gender and views on shield usage (χ^2^ = 6.59, df = 3, *p*-value = 0.086, Cramér’s V = 0.11); furthermore, no OLR was fitted.

**Figure 5:**
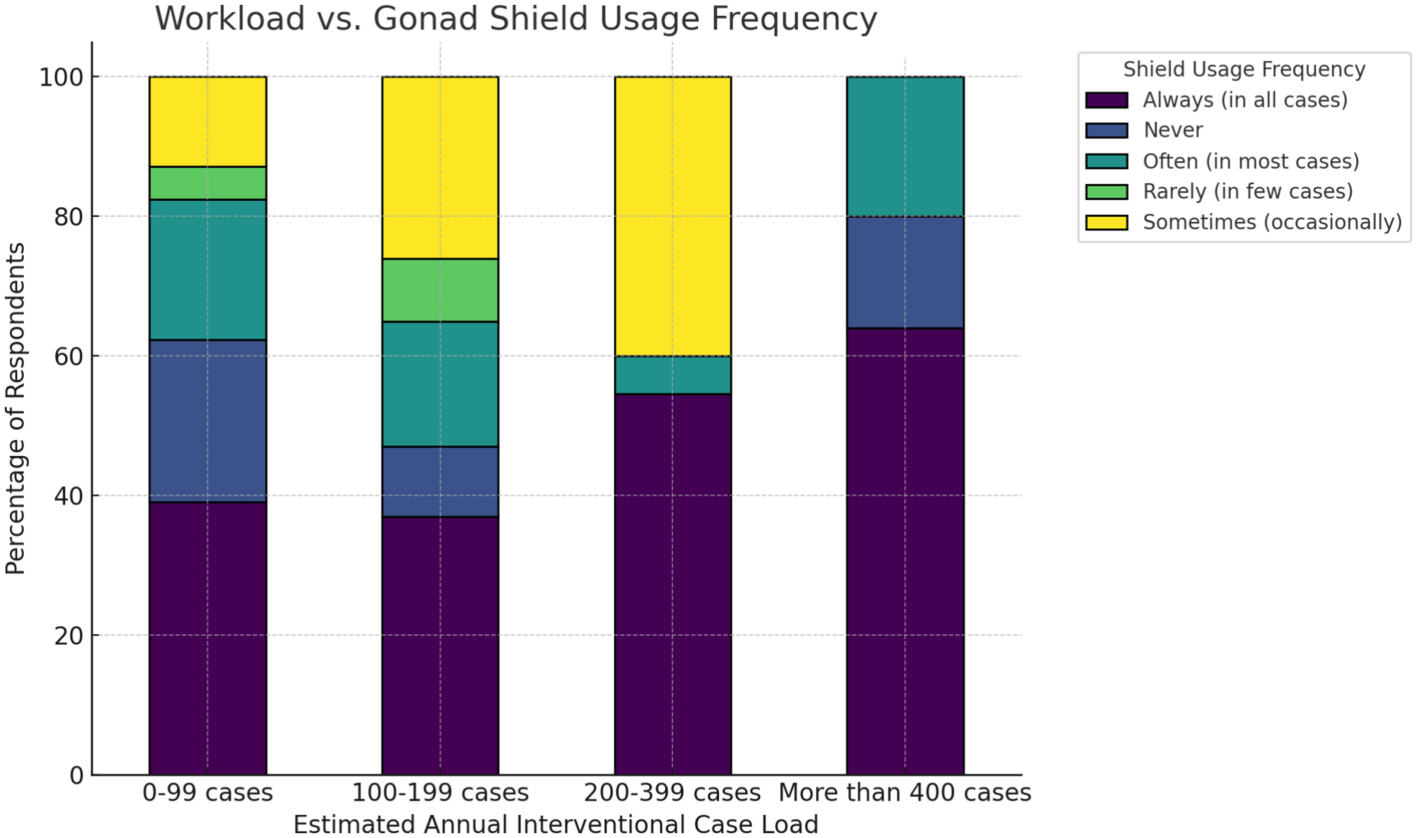
Stacked percentage bar chart of gonad shield usage by annual workload.

Considering the workload and use of the gonad shield, it is evident that workload was negatively linked to shield usage (Figure 5). Practitioners with higher annual caseloads (>200 cases/year) reported more consistent use of gonad shields, whereas those with lower volumes (0–99 cases/year) showed a greater variation in shield use, including more instances of non-use or occasional use. The chi-square test confirmed a statistically significant association between workload and shield usage frequency (χ^2^ = 85.27, df = 12, *p* = 4.03 × 10^−13^ (<0.05), Cramér’s V = 0.23). Ordinal logistic regression supported this pattern (Supplementary Figure S4).

**Figure 6:**
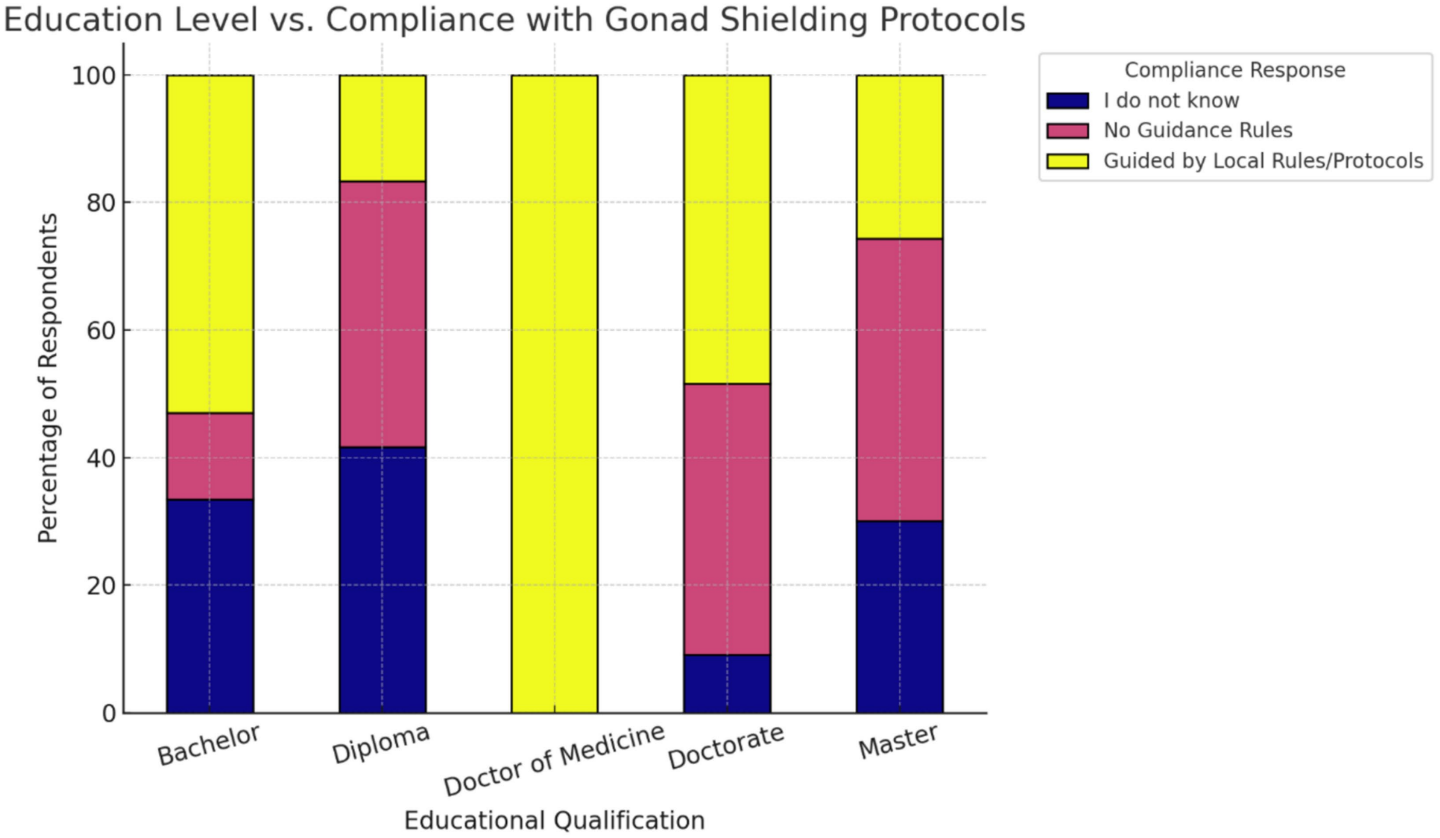
Stacked percentage bar chart of education level and protocol compliance.

Finally, we examined whether educational attainment was connected to protocol adherence. Bachelor’s degree holders exhibited the highest proportion reporting guided practice. In contrast, those with diplomas and master’s degrees showed a broader distribution, with a greater proportion selecting “No Guidance Rules” or “I do not know” (Figure 6). The chi-square test indicated a statistically significant relationship between education level and compliance behavior (χ^2^ = 73.82, df = 8, *p*-value = 8.5 × 10^−13^ (<0.05), Cramér’s V = 0.26). Counts for Doctor of Medicine were very small, so these proportions should be interpreted cautiously (Supplementary Figure S5).

## 4 Discussion

The most significant source of man-made radiation is medical imaging, which uses radiation for diagnosis and other purposes. IR is an emerging technology that enables accurate diagnosis and targeted therapy, thereby effectively enhancing patient care (23). The gonads are among the most sensitive organs to radiation and may be prone to stochastic risks [ICRP 103; (24)]. The main source of gonadal exposure for staff is the non-primary X-ray beam, which results from internal scatter radiation within the patient (11, 15, 25). Even when a lead apron is used, exposure to the gonads from scattered radiation during upper limb surgical procedures with C-arm fluoroscopy has been documented (26). Furthermore, radiation exposure to the gonads before conception may increase the incidence of hereditary effects (27, 28). Although wearing gonad shields can reduce gonadal exposure by up to 98% (29), staff use of gonad shields has been reported as occasional (30).

This cross-sectional multi-center study was designed to explore the level of awareness regarding the use of gonad shields during fluoroscopy-guided IR procedures.

According to practitioners’ feedback, years of experience were found to be inversely associated with the commitment to gonad shield usage (*p*-value < 0.05). Studies on the staff (with no consideration for the year of experience) reported a lack of significant commitment to the usage of radiation protection shielding (12, 14). A recent study has reported a similar inverse association of years of experience as a function of commitment to radiation protection practices (31), which was in agreement with our findings. Another study involving medical radiation workers in multi-unit facilities found a direct link between the two factors (32). Our study was conducted on IR unit workers, potentially clarifying the contradictions. Therefore, this finding suggests that educational and policy interventions should not only focus on early-career practitioners (Figure 1) but also on more experienced professionals.

Regarding the opinion of practitioners on the importance of gonad shield usage, the analysis of the feedback indicated a clear relationship between perceived importance and actual usage behavior (Figure 2). Practitioners who acknowledged the importance of shielding were more likely to use gonad shields consistently, while those who were unsure of its relevance exhibited minimal behavioral distinction from those who outright dismissed it. This implies that educational efforts must focus on reshaping underlying beliefs to effectively drive protective behaviors. This has also been observed in another hypothesis of the study, where radiation protection training was shown to play a formative role in enhancing adherence to protocols. Practitioners who received formal training were substantially more likely to comply with gonad shield usage guidelines (Figure 3). Thus, institutions should ensure that safety training is both structured and periodically refreshed to maximize retention and clinical application. This finding is in agreement with numerous studies (9, 10, 13, 16, 33, 34). Additionally, a study concerning the occupational radiation dose for IR staff demonstrated significant variation in radiation exposure to different body parts. Thus, a departmental protocol for radiation protection practices may be essential (35). In the gender-based analysis, it was shown that gender has no statistically significant effect on the use of gonad shields (*p* = 0.0.086). This finding has also been demonstrated in other studies (12, 14, 16, 31, 36, 37).

The workload and frequency of the gonad shield showed a linear relationship (Figure 5). A higher workload (>200 cases/year) may affect staff commitment to radiation protection practices. This may also explain the significant association observed in the second hypothesis, where practitioners’ opinions on the importance of gonad shield usage could reflect caseloads rather than their awareness of safety practices. Therefore, targeted educational intervention and training on shielding application may lead to improved consistency in the use of gonad protective shielding among practitioners (38).

Finally, a significant relationship between educational attainment and gonad shield protocol compliance was observed, where diploma- and master’s-level practitioners were less likely to adhere to the protocol compared to bachelor’s, while doctorate holders did not significantly differ. This non-linear association suggests that higher qualifications do not always translate into better radiation protection practices, potentially due to gaps between theoretical knowledge and adherence to clinical protocols (13, 39).

Consistent with the literature, the findings highlight the importance of education and structured training that could enhance compliance and address gaps in knowledge, suggesting that targeted and stratified training programs for various educational backgrounds could improve the alignment of radiation protection practices with institutional protocols. Thus, awareness and implementation of proper radiation protection practices are paramount to minimize the radiation risk and negative biological effects among practitioners on the IR unit (40). Additionally, a similar study could be conducted to examine staff’s perceptions of the recently published guidelines on the use of gonad shields (not only for patients).

This study has limitations. Its cross-sectional design restricts causative interpretations. Additionally, reliance on self-reported questionnaires introduces potential response bias. The findings are also geographically limited, which may affect their broader applicability. Future research should expand on these aspects by including longitudinal designs, broader sampling, and qualitative methods to deepen the understanding of radiation safety practices in IR units.

## 5 Conclusion

Interventional radiology staff views on gonad shields are inversely related to their years of practice but not to workload. Maintaining radiation protection training is crucial for better compliance with safety standards in interventional radiology. The impact of gender on gonad shield use was insignificant. Educational attainment was found to be associated with adherence to gonad shield protocols. Radiation protection protocols in interventional radiology are essential. This study could assist radiation safety officers in developing strategies to enhance staff adherence to safety regulations.

## Data Availability

The original contributions presented in the study are included in the article/Supplementary material; further inquiries can be directed to the corresponding author.
