# Supplementary material for: Knowledge, attitudes, and practices of radiology practitioners in Saudi Arabia toward the use of gonad shields during fluoroscopy-guided interventional radiography
Source: Front Med (Lausanne). 2025 Sep 25;12:1655457. doi: 10.3389/fmed.2025.1655457 (PMC12507816; doi:10.3389/fmed.2025.1655457)
Supplement: Supplementary file 1 [file Data_Sheet_1.pdf]

Supplementary File

Sample Size:

As per prior studies, awareness levels ranged widely, but we capped it at 50% [39], with a 5% margin of error. By using the following formula of Charan and Biswas [19].

$$sample\ size = \frac{Z^2 \times\ P(1-P)}{d^2}$$

Where, Z = 1.96 at P-value <0.05, P=0.5 and d = 0.05. The sample size should be 384 or more.

[19] J. Charan and T. Biswas, 'How to Calculate Sample Size for Different Study Designs in Medical Research?', *Indian J Psychol Med*, vol. 35, no. 2, pp. 121–126, Apr. 2013, doi: 10.4103/0253-7176.116232.

[39] R. Behzadmehr, M. Doostkami, Z. Sarchahi, L. Dinparast Saleh, and R. Behzadmehr, 'Radiation protection among health care workers: knowledge, attitude, practice, and clinical recommendations: a systematic review', *Rev Environ Health*, vol. 36, no. 2, pp. 223–234, Jun. 2021, doi: 10.1515/reveh-2020-0063.

Supplementary File

Tables:

Table S1: Participant demographics and professional characteristics (N = 527). Values are n (%); percentages use available-case denominators. Variables include gender, marital status, educational level, years of experience in IR, and annual interventional caseload. Category order follows a natural/ordinal progression. Abbreviation: IR, interventional radiology.

Variables	n	%
**Gender**		
Female	307	58.25%
Male	220	41.75%
**Total**	**527**	**100%**
**Marital Status**		
Married	151	28.65%
Single	376	71.34%
**Total**	**527**	**100%**
**Educational Level**		
Bachelor	389	73.81%
Diploma	24	4.55%
Doctor of Medicine	11	2.08%
Doctorate	33	6.26%
Master	70	13.28%
**Total**	**527**	**100%**
**Years of Experience**		
0 up to 4 years	349	66.22%
5 up to 9 years	75	14.23%
10 up to 15 years	70	13.28%
More than 15 years	33	6.26%
**Total**	**527**	**100%**
**Workload (IR Cases/Year)**		
0–99 cases	297	56.36%
100–199 cases	100	18.98%
200–399 cases	55	10.44%
More than 400 cases	75	14.23%
**Total**	**527**	**100%**

Supplementary File

Table S2: Mapping of study hypotheses to questionnaire items, variables measured and allowed responses (N = 527).

For each hypothesis, the table lists the predictor and outcome items from the final questionnaire, their allowed response options (ordered where applicable), and the primary analysis used. Gender was analyzed by χ^2 only because the outcome is nominal. Abbreviation: IR, interventional radiology.

Hypothesis	Predictor (questionnaire item)	Allowed responses (predictor)	Outcome (questionnaire item)	Allowed responses (outcome)	Primary analysis
H1. Experience affects gonad-shield usage	Years of experience in IR	0–4 years; 5–9 years; 10–15 years; >15 years	Shield-use frequency (Q2)	ORDERED: Never → Rarely (in few cases) → Sometimes (occasionally) → Often (in most cases) → Always (in all cases)	χ^2 (cross-tab); OLR (ref = 0–4 years)
H2. Belief in importance influences usage	Importance of protecting gonads (Q5)	I am not sure; No; Yes	Shield-use frequency (Q2)	ORDERED: Never → Rarely → Sometimes → Often → Always	χ^2; OLR (ref = "I am not sure")
H3. Training improves protocol compliance	Attended radiation-protection training (Q7)	I do not remember; No; Yes	Compliance with local guidance (Q10)	ORDERED: No rules → Don't know → Guided	χ^2; OLR (ref = "I do not remember")
H4. Gender influences views on who should be shielded	Gender	Female; Male	Views on who should be shielded (Q9)	NOMINAL: Should be used for all genders; Should be used for females only; Should be used for married persons only; Not to be used at all	χ^2 only (nominal outcome; OLR not applicable)
H5. Workload affects usage	Annual IR caseload	0–99 cases; 100–199 cases; 200–399 cases; >400 cases	Shield-use frequency (Q2)	ORDERED: Never → Rarely → Sometimes → Often → Always	χ^2; OLR (ref = 0–99 cases)
H6. Higher education relates to protocol compliance	Educational level	Diploma; Bachelor; Master; Doctorate; Doctor of Medicine	Compliance with local guidance (Q10)	ORDERED: No rules → Don't know → Guided	χ^2; OLR (ref = Bachelor)†

† Doctor of Medicine had very small counts and exhibited quasi-separation in OLR; interpret descriptively or consider collapsing categories in sensitivity analyses

Supplementary File

Figures of ordinal logistic regression:

(Fig.S1): Years of experience vs. frequency of gonad-shield use.

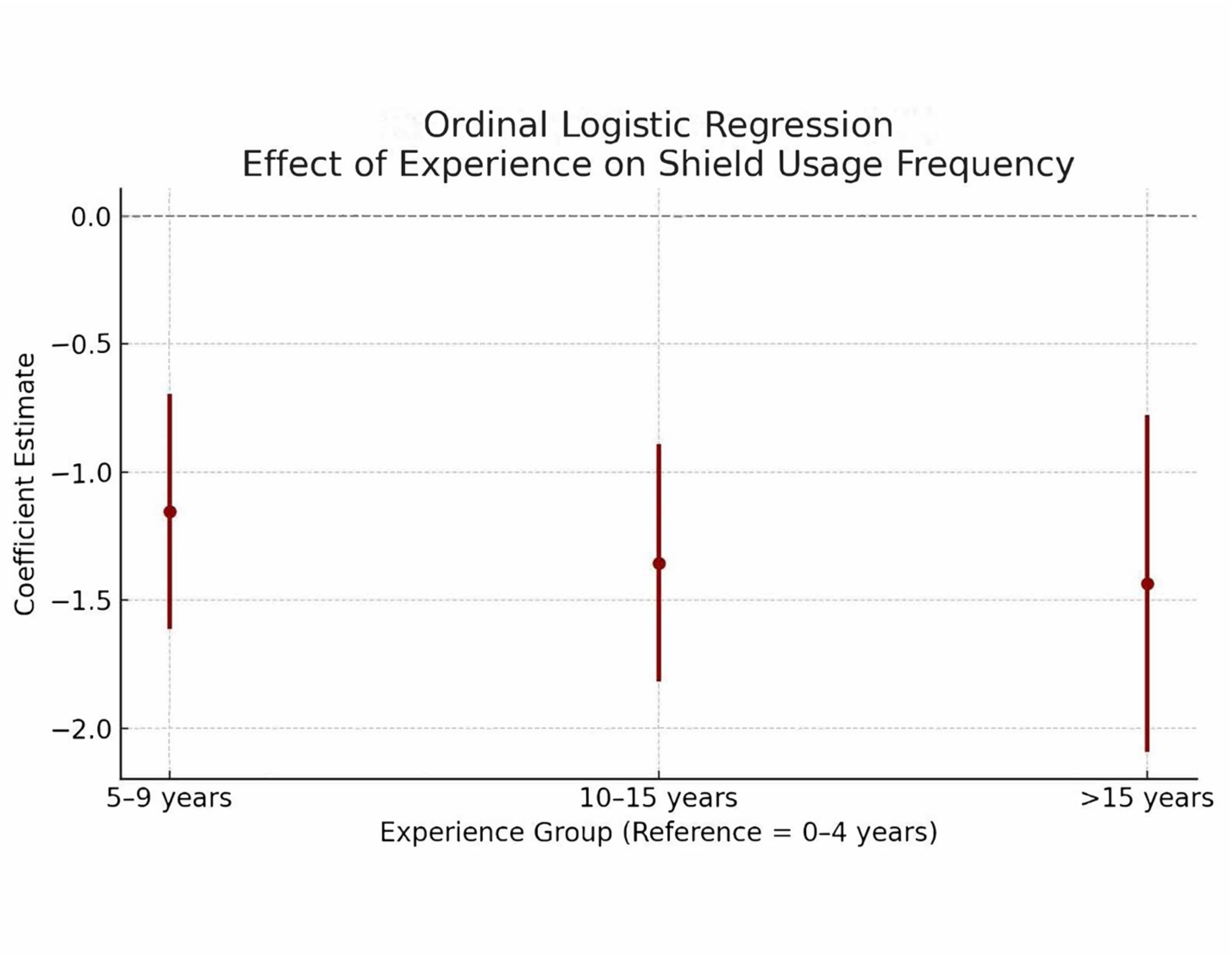

Proportional-odds logit model; outcome ordered Never < Rarely < Sometimes < Often < Always (higher = more frequent use). Reference = 0–4 years. Compared with the reference, practitioners with 5–9 years had lower odds of more frequent use (OR 0.49, 95% CI 0.32–0.76, $p = 0.0012$), 10–15 years: OR 0.39 (0.25–0.61, $p = 4.7\times10^{-5}$), and >15 years: OR 0.21 (0.11–0.39, $p = 1.0\times10^{-6}$).

Supplementary File

(Fig.S2): Perceived importance of protecting gonads vs. frequency of use.

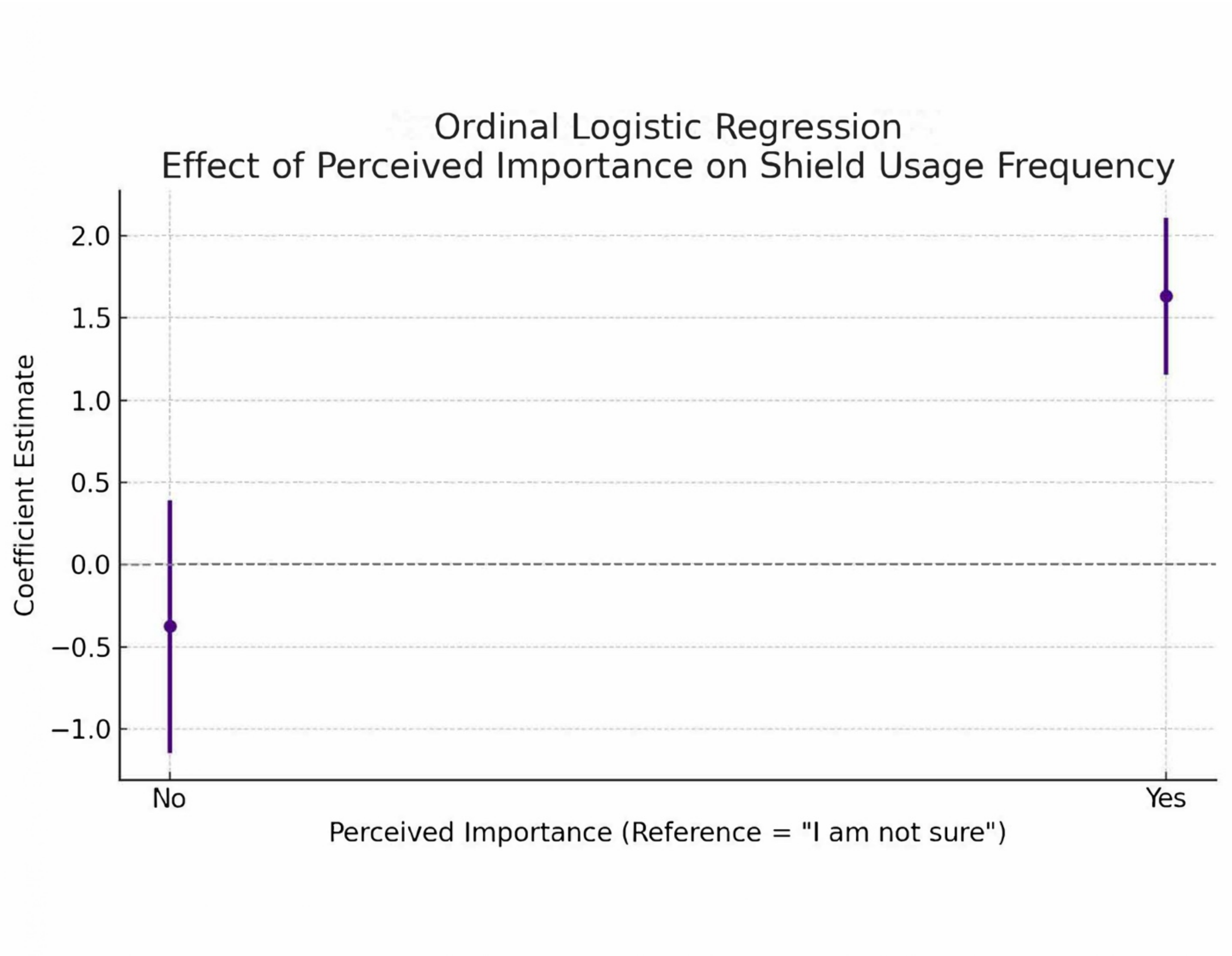

Proportional-odds logit model; outcome ordered Never + Always. Reference = “I am not sure.” Rating shielding as important was associated with higher odds of more frequent use (OR 5.11, 95% CI 3.17–8.24, $p = 2.1\times10^{-11}$); “No” did not differ from “not sure” (OR 0.69, 95% CI 0.32–1.48, p = 0.336).

Supplementary File

(Fig.S3): **Training attendance vs. protocol compliance.**

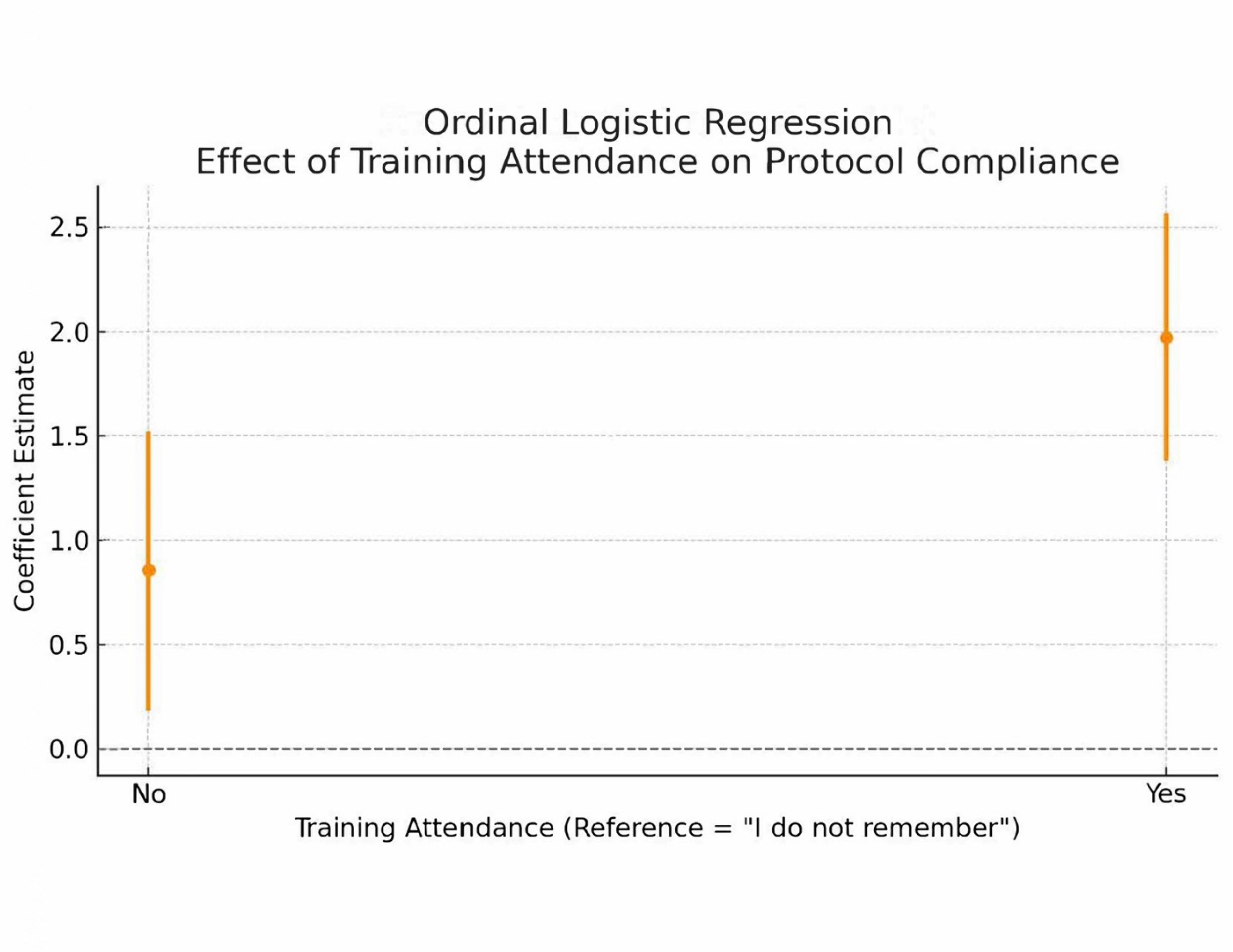

Proportional-odds logit model; compliance ordered No rules < Don’t know < Guided (higher = greater compliance). Reference = “I do not remember.” No training: OR 1.47 (95% CI 0.84–2.57, $p = 0.181$) (ns); Yes (trained): OR 2.66 (1.63–4.33, $p = 8.8\times10^{-5}$).

Supplementary File

(Fig.S4): Annual workload vs. frequency of use.

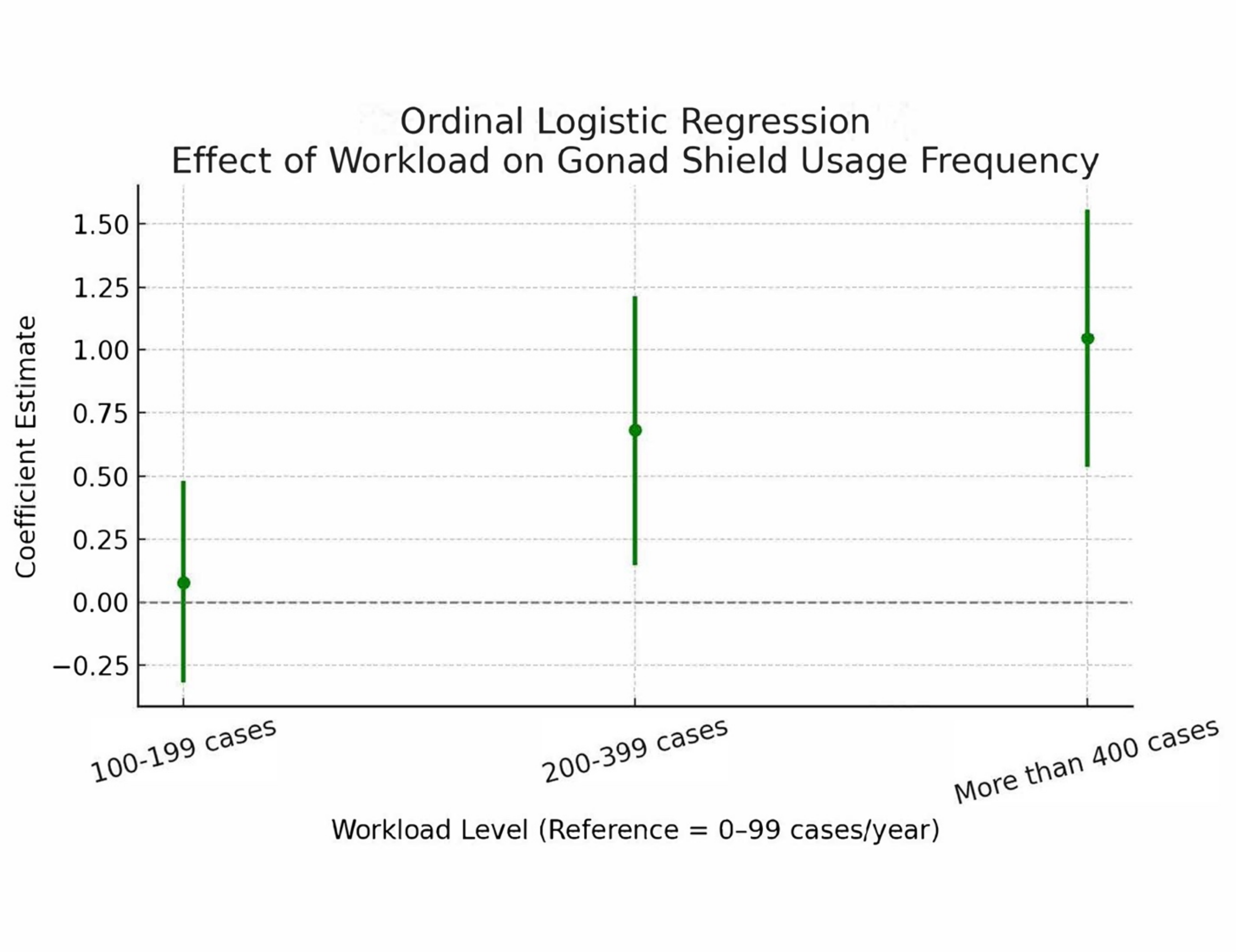

Proportional-odds logit model; outcome ordered Never + Always. Reference = 0–99 cases/year. 100–199 cases: OR 1.08 (95% CI 0.73–1.61, $p = 0.697$); 200–399 cases: OR 1.98 (1.16–3.38, $p = 0.0123$); >400 cases: OR 2.85 (1.71–4.75, $p = 5.7\times10^{-5}$).

Supplementary File

(Fig.S5): Educational level vs. protocol compliance.

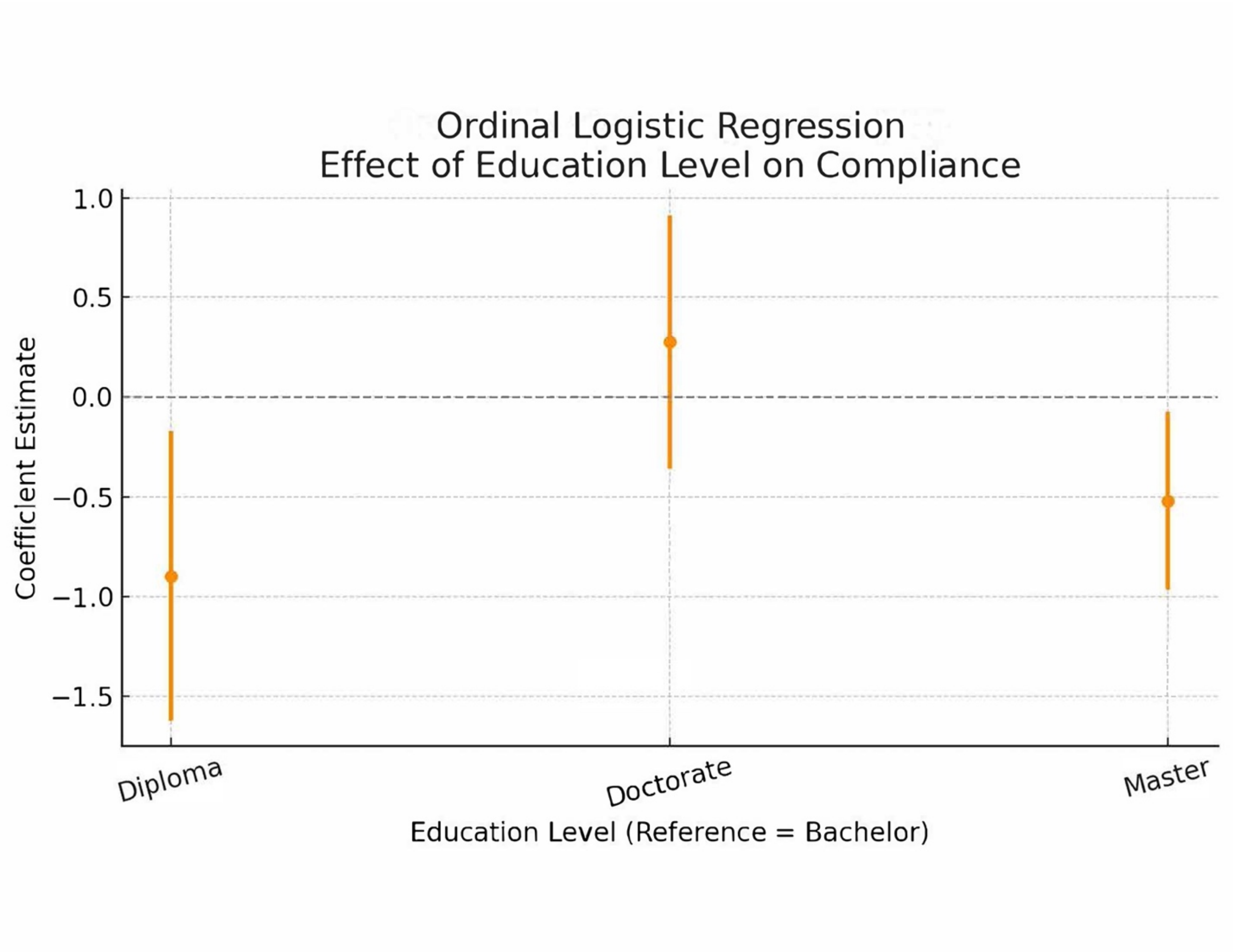

Proportional-odds logit model; compliance ordered No rules < Don't know < Guided. Reference = Bachelor. Diploma: OR 0.23 (95% CI 0.11–0.48, $p = 1.19\times10^{-4}$); Master: OR 0.25 (0.15–0.41, $p = 3.42\times10^{-8}$); Doctorate: OR 0.47 (0.23–0.98, p = 0.0427). Doctor of Medicine showed quasi-separation (very small n); coefficient unstable report as descriptive only.
